# Liver clear cell foci and viral infection are associated with non-cirrhotic, non-fibrolamellar hepatocellular carcinoma in young patients from South America

**DOI:** 10.1038/s41598-018-28286-0

**Published:** 2018-07-30

**Authors:** Luis Cano, Juan Pablo Cerapio, Eloy Ruiz, Agnès Marchio, Bruno Turlin, Sandro Casavilca, Luis Taxa, Guillaume Marti, Eric Deharo, Pascal Pineau, Stéphane Bertani

**Affiliations:** 1 Université de Rennes 1, INSERM, CNRS, U 1241 NUMECAN, Rennes, France; 2 Sorbonne Universités, UPMC Univ Paris 06, Institut Pasteur, Unité Organisation Nucléaire et Oncogenèse, Paris, France; 3 Instituto Nacional de Enfermedades Neoplásicas, Departamento de Cirugía en Abdomen, Lima, Peru; 4 Institut Pasteur, Unité Organisation Nucléaire et Oncogenèse, INSERM, U 993, Paris, France; 5 Instituto Nacional de Enfermedades Neoplásicas, Departamento de Patología, Lima, Peru; 6 Université de Toulouse, IRD, UPS, UMR 152 PHARMADEV, Toulouse, France

## Abstract

We previously described a divergent clinical and molecular presentation of hepatocellular carcinoma (HCC) in Peru. The present study aimed to further characterize the tissue features associated with this singular nosological form of HCC in order to gain insight into the natural history of the disease. We performed an exploratory analysis of the histology of both tumor and non-tumor liver (NTL) tissues from 50 Peruvian HCC patients, and compared with that of 75 individuals with non-HCC liver tumor or benign liver lesions as a baseline for NTL features. We complemented this approach with a transcriptome analysis in a subset of NTL tissue samples and also performed an ultra-sensitive hepatitis B virus (HBV) detection in liver tissues of the patients. Overall, results highlighted the low rate of liver parenchymal alterations in a young patient cohort (median age: 40 years old), despite a strong prevalence of underlying HBV infection (c. 67%). Withal, liver clear cell foci of cellular alteration were genuinely associated with HCC and appended to some changes in immune and G protein-coupled receptor gene expression ontologies. Our findings confirm the occurrence of a particular setting of HCC in South America, a region where the pathophysiology of liver cancer remains largely unexplored.

## Introduction

The development of hepatocellular carcinoma (HCC) is frequently associated with a chronic insult of the liver and long-lasting hepatic diseases that trigger over time the induction of a carcinogenic process^1–3^. One of the prevailing patterns of the natural history of the disease is the string of pathophysiological events driving liver disorders, such as chronic liver inflammation and steatohepatitis, towards hepatic fibrosis^4^. Liver cirrhosis represents often the final stage of this sequence that drastically enhances the risk of developing HCC^5^. Thus, a larger number of HCC cases described hitherto are found in cirrhotic patients, whereas non-cirrhotic liver tumor presentation is more rarely described.

We previously described a peculiar clinical epidemiology of HCC among patients attending the National Cancer Institute of Peru (INEN) in Lima, Peru^6^. These HCC patients displayed notable clinical features: (i) 50% of them were relatively young with a median age below 40 years old; (ii) the large majority of the patients presented with advanced-stage HCC and large tumors exceeding 10 cm diameter; and last but not least, (iii) only 11% of HCCs occurred in the context of cirrhosis, whereas the proportion of cirrhotic patients with HCC in relevant literature chiefly ranges from 80% to 90%^7–9^. We then consolidated this peculiar clinical presentation at the molecular genetic level with a unique mutation spectrum, in which the major class of alterations was epitomized by genomic short indels^10^. This mutation spectrum is not only unprecedented for HCC, but also unique among solid tumors in which indels are a marginal subset of genetic alterations^11,12^. Thus, while consistent detection of advanced clinical stages of HCC may reflect both the lack of screening program and a deficient access to health care in Peru, the situation encountered in the country is also most likely due to some biological features intrinsic to the natural history of the disease in the population of Peru, and more broadly from South America^13^.

As the very large majority of the Peruvian HCC patients attending INEN intriguingly do not present with full-fledged cirrhosis, we decided to determine, from a histological point of view, the detailed pathological status of the non-tumor liver (NTL) parenchyma of these patients. To address this issue, we performed a comprehensive study examining retrospectively liver sections from a cohort that complied with the HCC patient population structure from INEN in terms of gender and age distribution. In parallel, we contrasted this HCC patient population with a second cohort of Peruvian individuals with primary or secondary, non-HCC liver tumor or benign liver lesions as a baseline for NTL features in order to gain insight into the tissue alterations associated with the development of non-fibrolamellar HCC in young, non-cirrhotic individuals.

## Results

Details of the study are presented extensively in the Supplementary Information file.

### Clinical demography

**Figure 1:**
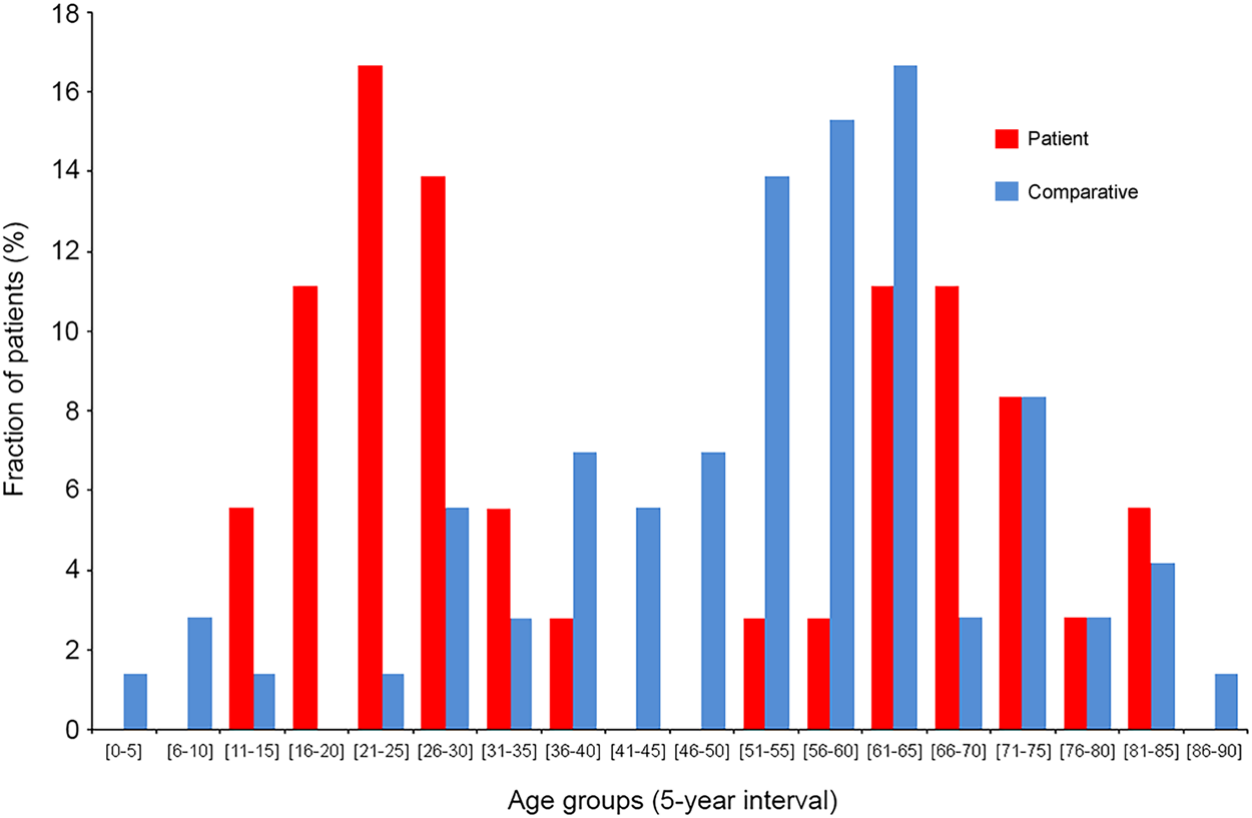
Age distribution of both Patient Group and Comparative Group. Histogram shows the age distribution of HCC patients (red) and comparative individuals (blue). X-axis shows age (5-year interval); Y-axis shows percentage of HCC patients (n = 50) and comparative individuals (n = 75) for a given age group.

Table 1 shows an overview of the demographical, clinical features of the 50 patients with HCC (Patient Group) included in the present study. The mean and median ages of the Patient Group were respectively 46.5 ± 20.9 and 40 years old, ranging from age 10 to age 96. The structure of the Patient Group regarding age was fitting with the bimodal distribution previously described among the patient population with HCC in Peru, displaying 52% of individuals below or equal to age 40 (n = 26) and 48% of individuals above age 40 (n = 24) (Fig. 1)^6,10,14–16^. The ratio of males to females was 1.5 with 60% of men (n = 30) and 40% of women (n = 20), once again, in accordance with the clinical epidemiological pattern reported hitherto for HCC in Peru. Chronic infection with hepatitis B virus (HBV) was found in 52% of HCC patients (n = 26), whereas only one patient was recorded positive for infection with hepatitis C virus (HCV). Overall, the average level of alpha-fetoprotein (AFP) in serum was exceedingly elevated with 76,255.9 ± 140,085.6 ng/mL (median 5,286.5 ng/mL), which is a persistent hallmark in younger Peruvian HCC patients^6^.

**Table 1:** Baseline demographical and clinical features of the Peruvian HCC patients.

Feature	Parameter	Number	Percentage
Cohort	Patient	50	100
Age (years)	Mean ± s.d.	46.5 ± 20.9	n/a
	Median	40	n/a
	Range	[10–96]	n/a
	Interquartile range	38.5	n/a
Gender	Female	20	40
	Male	30	60
HBsAg	Negative	23	46
	Positive	26	52
Anti-HCV	Negative	49	98
	Positive	1	2
Tumor size (cm)	Mean ± s.d.	13.9 ± 6.2	n/a
	Median	14	n/a
	Range	[2.5–29]	n/a
	Interquartile range	7.6	n/a
	Less than 5 cm	3	6
	Between 5 and 10 cm	12	24
	More than 10 cm	35	70
Tumor nodule	Single	46	92
	Multinodular	4	8
Vascular invasion	Absent	38	76
	Macro	4	8
	Micro	3	6
	Macro and micro	5	10
Tumor grade	G1	14	28
	G2	24	48
	G3	12	24
Tumor growth pattern	Acinar	2	4
	Compact	2	4
	Mixed growth pattern	19	38
	Trabecular	27	54
AFP (ng/mL)	Mean ± s.d.	76,255.9 ± 140,085.6	n/a
	Median	5,286.5	n/a
	Range	[1–481,050]	n/a
	Interquartile range	72,455.7	n/a

Percentages are expressed as a ratio of the 50 patients investigated for the considered parameter. Tumor architecture and grading (G1–G4) were defined according to established classifications^50,51^. Mean values are presented with ±standard deviation (s.d.). AFP, alpha-fetoprotein; HBsAg, HBV surface antigen; HCV, hepatitis C virus; n/a, not applicable.

A second group consisting of 75 individuals with primary or secondary, non-HCC liver tumor or benign liver lesions (Comparative Group) was included in the design of the study in order to approximate a baseline for NTL features among Peruvian people who underwent a hepatectomy. The ratio of males to females in the Comparative Group was 0.8 with 44% of men (n = 33) and 56% of women (n = 42). The mean and median ages of the Comparative Group were respectively 57.6 ± 17.7 and 60 years old, ranging from age 6 to age 90 (Fig. 1). Intra-hepatic neoplasms from the Comparative Group included metastatic colorectal cancer (n = 39); gallbladder cancer (n = 10); breast cancer (n = 6); neuroendocrine tumor (n = 5); renal cancer and undifferentiated tumor (n = 2); and angiosarcoma, gastrointestinal stromal tumor, leiomyosarcoma, diffuse large B-cell lymphoma, and melanoma (all n = 1). In addition, Comparative Group also included individuals with benign liver lesions such as chronic liver inflammation (n = 2) and primary sclerosing cholangitis, focal nodular hyperplasia, peliosis hepatis, and inflammatory pseudotumor of the liver (all n = 1).

### Clinical presentation of hepatocellular carcinoma

The mean diameter of HCCs was 13.9 ± 6.2 cm with a median of 14 cm diameter (Table 1). Tumors were well differentiated (G1) in 28% of the cases (n = 14), moderately differentiated (G2) in 48% of the cases (n = 24), and poorly differentiated (G3) in 24% of the cases (n = 12); no tumor was graded undifferentiated (G4). The histological growth pattern of HCC was trabecular in 54% of the cases (n = 27), mixed in 38% (n = 19), compact in 4% (n = 2), and acinar in 4% (n = 2). These features were in accordance with those reported hitherto^14^. Classical and steatohepatitic histological types represented the very large majority of the HCCs scrutinized with 56% (n = 28) and 42% (n = 21), respectively. A single fibrolamellar carcinoma variant (2%) was observed. Macro- and/or micro-vascular invasion was observed in 24% of the cases (n = 12), and 28% of the patients had developed distant metastasis (n = 14).

### Histology of the non-tumor liver parenchyma

Table 2 presents descriptive statistical results for hepatic fibrosis staging in both the Patient and Comparative Groups. In the Patient Group, the large majority of the NTLs, i.e. 72%, presented with null to moderate Stages 1 and 2 fibrosis (n = 36). In addition, 12% of the NTLs were recorded at Stage 3 fibrosis (n = 6); and 16% displayed cirrhosis, i.e. eight patients including merely two males less than 50 years of age. There was no statistical trend towards higher fibrosis stage in the Patient Group compared to the Comparative Group (*P* = 6.20E-02), using the Cochran–Armitage test for trend.

**Table 2:** Fibrosis staging of non-tumor liver parenchyma from both Peruvian patients with hepatocellular carcinoma and comparative individuals.

Fibrosis stage	Contingency in Patient Group (n)	Percentage in Patient Group (%)	Contingency in Comparative Group (n)	Percentage in Comparative Group (%)
Stage 0	17	34	33	44
Stage 1	14	28	21	28
Stage 2	5	10	11	14.6
Stage 3	6	12	6	8
Stage 4	8	16	4	5.4
Total	50	100	75	100

Scheuer staging system was used to determine the stage of hepatic fibrosis^54^. Stage 0, no fibrosis and normal amount of connective tissue; Stage 1, enlarged, fibrotic portal tracts; Stage 2, periportal or portal-portal septa, but intact architecture; Stage 3, bridging fibrosis with architectural distortion, but no obvious cirrhosis; Stage 4, probable or definite cirrhosis.

About 26% of HCC patients (n = 14) and 30.7% of comparative individuals (n = 23) displayed macro- and micro-vesicular hepatic steatosis that, in any case, did not exceed 50% of parenchymal involvement (Table 3). There was no statistical trend towards a higher grade of steatosis in the Patient Group compared to the Comparative Group (*P* = 0.28). Lobular activity was slightly significantly contrasted with a trend toward mild inflammation in HCC patients (*P* = 4.70E-02); whereas the portal/periportal inflammation level was not statistically different between both groups (*P* = 0.30) (see Supplementary Table S1). Definite steatohepatitis was scored in 8% of HCC cases (n = 4) and 6.7% of the comparative individuals (n = 5). Once again, there was no significant difference regarding fatty liver changes between both groups (*P* = 1.00).

**Table 3:** Steatosis grading of non-tumor liver parenchyma from both Peruvian patients with hepatocellular carcinoma and comparative individuals.

Steatosis grade	Contingency in Patient Group (n)	Percentage in Patient Group (%)	Contingency in Comparative Group (n)	Percentage in Comparative Group (%)
Grade 0	37	74	52	69.3
Grade 1	11	22	13	17.3
Grade 2	1	2	8	10.7
Grade 3	1	2	2	2.7
Total	50	100	75	100

Hepatic steatosis was graded using the system established by the Non-alcoholic Steatohepatitis Clinical Research Network^52^. Grade 0, less than 5% of parenchymal involvement by steatosis; Grade 1, from 5% to 33% of parenchymal involvement by steatosis; Grade 2, from 33% to 66% of parenchymal involvement by steatosis; Grade 3, more than 66% of parenchymal involvement by steatosis.

Table 4 details the descriptive statistical results for ferric iron overload in NTL in both the Patient and Comparative Groups. A certain degree of parenchymal siderosis was recorded in half of the HCC-associated NTLs with 50% of the samples (n = 25) scoring in histological Grades 1+ to 4+. On the same hand, 40% of the NTLs from the Comparative Group (n = 30) scored histological Grades 1+ to 4+. As a result, no differential trend in iron accumulation was statistically revealed between the Patient Group and the Comparative Group (*P* = 0.58).

**Table 4:** Histological grading of ferric iron overload in non-tumor liver parenchyma from both Peruvian patients with hepatocellular carcinoma and comparative individuals.

Iron overload grade	Occurrence in Patient Group (n)	Percentage in Patient Group (%)	Occurrence in Comparative Group (n)	Percentage in Comparative Group (%)
Grade 0	25	50	45	60
Grade 1+	9	18	9	12
Grade 2+	8	16	11	14.7
Grade 3+	6	12	6	8
Grade 4+	2	4	4	5.3
Total	50	100	75	100

Ferric iron accumulation was graded using the system established by LeSage and colleagues^55^. Grade 0, no stainable iron; Grade 1+, less than 25% of hepatic cells positive; Grade 2+, between 25% and 50% of hepatic cells positive; Grade 3+, between 50% and 75% of hepatic cells positive; Grade 4+, more than 75% of hepatic cells positive.

### Data mining and exploratory multivariate data analysis

**Figure 2:**
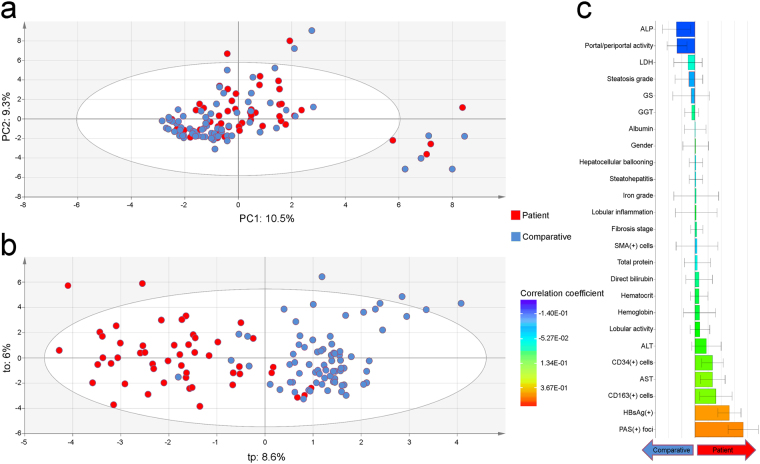
Exploratory multivariate data analysis of non-tumor liver features in both Peruvian patients with hepatocellular carcinoma and comparative individuals. (**a**) Unsupervised PCA score plot (PC1: 10.5% versus PC2: 9.3%) displaying a blended assemblage of HCC patients and comparative individuals. The scattered cluster located outside the Hotelling’s T^2^ limits on the lower right corner plots individuals with hepatocellular ballooning and steatohepatitis. (**b**) OPLS-DA score plot for the first predictive (tp: 8.6%) versus orthogonal (to: 6%) components showing dissociation between HCC patients and comparative individuals. The performance of the prediction model was satisfactory (R2Y = 0.68; Q2Y = 0.53). (**a**,**b**) HCC patients and comparative individuals are plotted in red and blue, respectively. (**c**) Regression coefficient plot for the OPLS-DA model with biomedical features of both HCC patients and comparatives individuals. Right coefficient boxes indicate a correlation with Patient Group and left coefficient boxes a correlation with Comparative Group; coefficient box color gives an indication about the degree of correlation; and error bars represent the Jack-knife standard error of the Y loading weight vector computed from the rounds of cross validation.

Principal component analysis (PCA) was applied as an exploratory data analysis to provide an unsupervised overview of clinical and histological data. No discrimination was highlighted between the Patient Group and the Comparative Group on the PCA score plot (Fig. 2a). Some people were characterized by the prevalence of hepatocellular ballooning and steatohepatitis, but this subset of individuals was indistinctively found in both groups. Orthogonal projection to latent structure-discriminant analysis (OPLS-DA) was then employed to disjoin HCC patients from comparative individuals (Fig. 2b). According to coefficient plot, there was no dramatic difference between the Patient Group and the Comparative Group for most clinical, biochemical, and histological features scrutinized, with the notable exceptions of periodic acid–Schiff (PAS) positive [PAS(+)] hepatic foci of cellular alteration (*P* = 4.46E-13) and HBV surface antigen (HBsAg) carriage (*P* = 1.06E-08), as well as, albeit to a significantly lesser extent, macrophage (CD163) and hematopoietic stem cell (CD34) counts (*P* = 6.43E-06 and 3.31E-05, respectively) (Fig. 2c) (see Supplementary Fig. S1a,b and Supplementary Table S2).

### Histological features of the hepatic foci of cellular alteration

**Figure 3:**
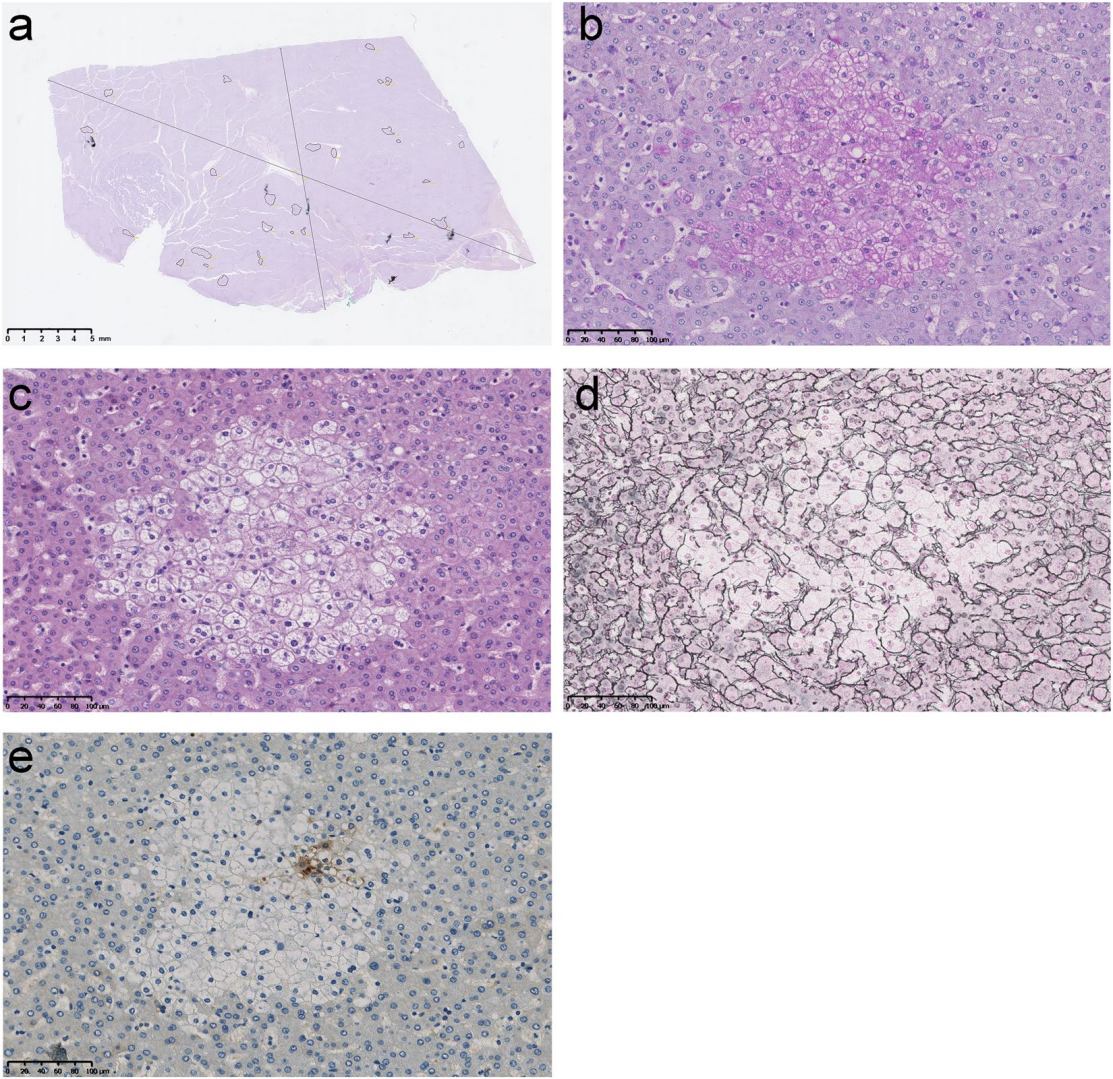
Illustration of the hepatic foci of cellular alteration observed in non-tumor liver parenchyma of Peruvian patients with hepatocellular carcinoma. (**a**) NTL section from a 37-year-old Peruvian male individual with a 16-cm-diameter well differentiated (G1), trabecular growth pattern HCC. The surrounded areas indicate location and size of liver clear cell foci within the section. NTL section dimension; 25.5 mm-length, 15.1 mm-width. Scale bar; 5 mm. (**b–e**) Representative features in a series of serial NTL sections from a 32-year-old Peruvian male individual with a 14-cm-diameter moderately differentiated (G2), trabecular growth pattern HCC under medium power magnification (30x). Scale bars; 100 µm. (**b**) A hepatic focus of cellular alteration under PAS staining. This histological feature was significantly associated with Patient Group by OPLS-DA (Fig. 2b). (**c**) The same focus than in (b) under hematoxylin–eosin staining. The focus is displaying a clear cell-like appearance. (**d**) The same focus than in (b,c) under Gömöri reticulin staining. It is observed a distortion of the trabecular architecture over the focal area. (**e**) GS immunohistochemistry on the same focus than in (b–d). GS expression is observed heterogeneously in some cells of the hepatic focus of cellular alteration.

As pointed out by OPLS-DA, much more hepatic foci of cellular alteration were strongly marked positively by PAS staining in NTL sections of Peruvian HCC patients, in a significant manner [62% (n = 31) in Patient Group versus 4% (n = 3) in Comparative Group; *P* = 4.46E-13] (Fig. 2c). When scored positive, these foci were found abundantly in liver parenchyma with a density of 2.19 ± 2.37 ind./cm^2^ in NTL section and were preferentially observed in Zones 2 and 3 of the hepatic acinus (Fig. 3a). Under PAS staining, these foci featured a massive accumulation of cytoplasmic glycogen storage compared to the adjacent parenchymal hepatocytes (Fig. 3b). While being observed under hematoxylin–eosin staining, these clear cell foci were well defined and presented a disorganized rearrangement of the hepatic plates in a parenchymal area of 0.14 mm^2^ on average, with a distortion of the trabecular architecture (Fig. 3c,d). Within a focus, cells were reduced in size compared to surrounding hepatocytes. They exhibited an altered nuclear-cytoplasmic ratio with a centrally positioned nucleus and cytoplasm with pale eosinophilic staining, without intracytoplasmic inclusions. A discrete inflammation was monitored within the nearby perisinusoidal spaces. In addition, it could be observed some steatotic hepatocytes in the surrounding tissue microenvironment, but this was not always consistent. It was registered some degree of *in situ* congruence between these foci and focal glutamine synthetase (GS) expression found in NTL parenchyma of Peruvian HCC patients (Fig. 3e). No statistical correlation was observed between PAS(+) and the prevalence of steatosis (*P* = 0.19) and steatohepatitis (*P* = 0.28).

### Differential gene expression analysis

**Figure 4:**
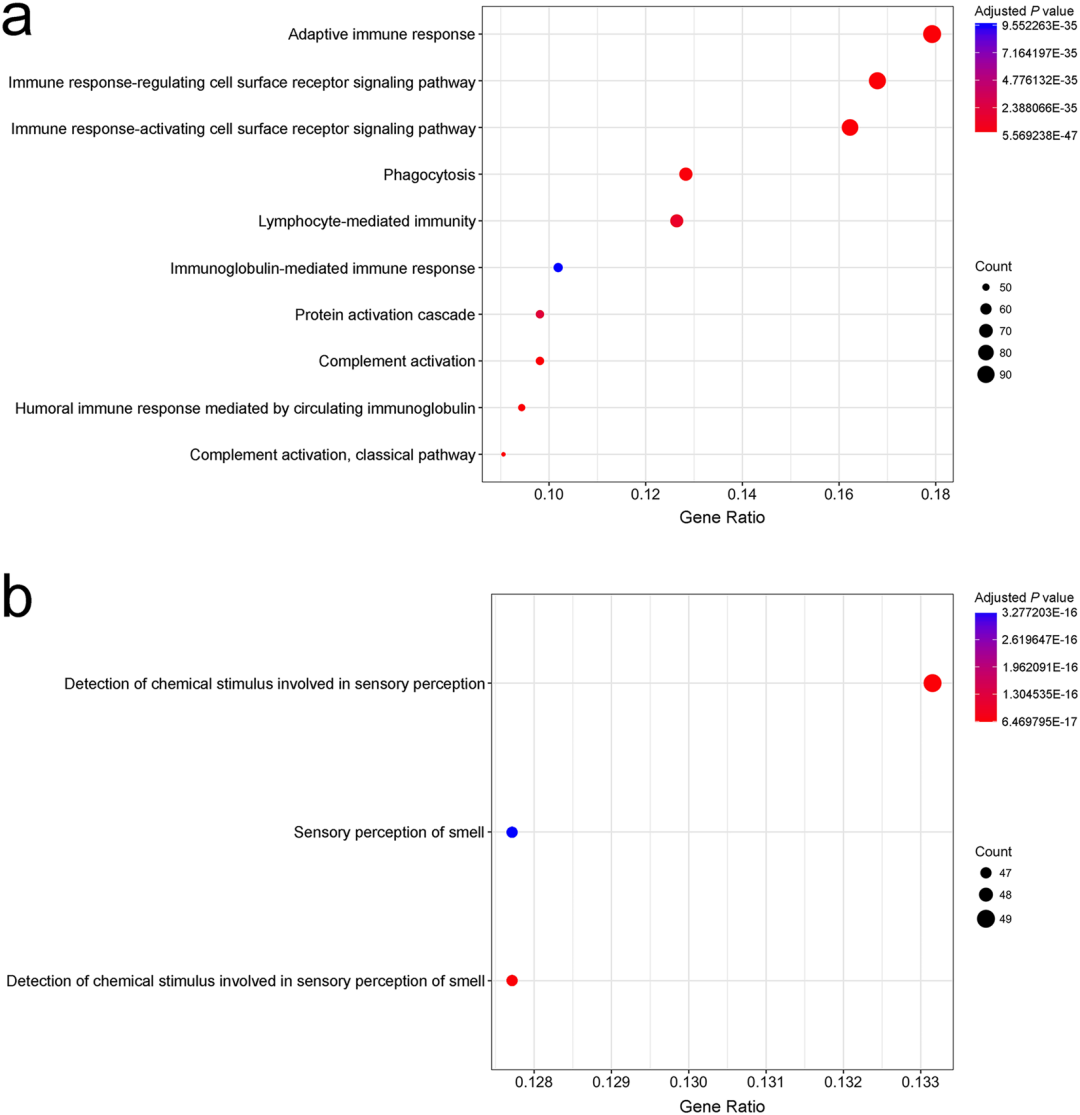
Gene ontology enrichment analysis on expression dataset of non-tumor livers with and without PAS positive hepatic foci of cellular alteration. (**a**) Point plot representation of the ontologies downregulated in PAS(+) NTL compared to PAS(−) NTL of Peruvian HCC patients. (**b**) Point plot representation of the ontologies upregulated in PAS(+) NTL compared to PAS(−) NTL of Peruvian HCC patients. (**a**,**b**) X-axis shows Gene Ratio of each term and Y-axis shows the Gene Ontology pathway terms; point size scales the gene count; and point color illustrates the adjusted *P* value.

Gene expression profiling was performed in a subset of NTLs from individuals of the Patient Group for whom we had high quality RNA, i.e. six PAS(+) patients and three patients with no such hepatic lesion [PAS(−)] (see Supplementary Table S1). Interestingly, differential analysis revealed contrasts in gene expression between PAS(+) and PAS(−) NTLs. At first sight, the immune status of PAS(+) NTLs appeared to be altered at the transcriptional level compared to PAS(−) NTLs, as the gene expression of multiple immune-related ontologies were significantly mitigated (Fig. 4a). Conversely, some genes of the olfactory receptor superfamily were significantly overexpressed in PAS(+) NTLs compared to PAS(−) NTLs (Fig. 4b). In addition, we purposely focused on genes involved in carbohydrate metabolic process, as PAS stains tissue structures containing a high proportion of carbohydrates, e.g. glycogen, glycoproteins, and proteoglycans^17^. We found that several genes, which are key players for carbohydrate anabolism, were transcriptionally overexpressed in PAS(+) NTLs compared to PAS(−) NTLs, such as insulin-like growth factor 1 (*IGF1*) (*P* = 6.17E-03), insulin receptor substrate 1 (*IRS1*) (*P* = 2.26E-02), forkhead box O1 (*FOXO1*) (*P* = 1.11E-02), or even pyruvate dehydrogenase kinase 4 (*PDK4*) (*P* = 1.73E-03) (see Supplementary Table S3). However, other genes involved in carbohydrate catabolism, like galactosidase alpha (*GLA*), were downregulated (*P* = 4.95E-02). Finally, *SERPINA1* gene linked to alpha–1–antitrypsin deficiency was not statistically differentially expressed between PAS(+) and PAS(−) NTLs (*P* = 0.96), whereas differential transcription of few other genes of the serine proteinase inhibitor (serpin) superfamily, i.e. *SERPIND1*, *SERPINE1*, and *SERPINF2*, were monitored (*P* = 3.55E-02, 3.88E-02, and 4.62E-02, respectively) (see Supplementary Table S3).

### Evaluation of the burden of hepatitis B virus in tumor and non-tumor liver tissues

As revealed by OPLS-DA, HBsAg carriage was significantly found in 52% of HCC patients (n = 26), whereas merely 6.7% comparative individuals were recorded positive for HBV infection (n = 5) (*P* = 1.06E-08). A comprehensive molecular detection of HBV was then performed in a subset of 30 HCC and parent NTL matched pairs for which we had total DNA extracted from fresh frozen tissues. In this sub-cohort, prevalence of HBsAg carriage was 43.3% (n = 13). The presence of HBV DNA was monitored in at least one sample of the matched pairs of liver tissues in 66.7% of the cases (n = 20), which implies that 23.3% of HBV DNA-positive patients was initially monitored negative for HBsAg serology (n = 7) (see Supplementary Table S1). Total HBV DNA burden was very low with median values of 6.01E-01 viral genome equivalent copy per cell in HCC and 2.89E-01 in NTL, with no statistical difference between the two types of tissues (*P* = 0.08) (see Supplementary Fig. S2). Likewise, no correlation was established between prevalence of HBsAg carriage and/or HBV DNA in NTL with PAS(+) NTL (*P* = 0.70).

## Discussion

HCC arising in non-cirrhotic liver displays several histopathological and clinical features that distinguish it from HCC occurring in the setting of cirrhosis^18^. Epidemiologically, the age distribution of non-cirrhotic HCC patients delineates a bimodal pattern, with two peaks at the third and the seventh decades^18,19^. We previously reported this very specific incidence in Peru where non-cirrhotic individuals represent the vast majority of HCC patients attending INEN and age distribution displays bimodality with two maximums at age 25 and age 64 (Fig. 1 and Table 1)^6^. This peculiar clinical epidemiology is associated with a singular mutation spectrum, in which has recorded a low rate of alterations in HCC-related gene mutation hotspots and short indels as the prominent class of genetic defects^10^. This has some implications for the clinical management strategy pertaining to HCC patients with no cirrhosis in Peru and elsewhere^13,14,20^. Despite this intriguing clinicopathological presentation of the tumor, no study had been conducted so far to precisely characterize the histopathological status of NTL from Peruvian patients with HCC. Hence, we assembled a cohort of HCC patients attending INEN that matched the structure of the Peruvian population of HCC patients both in terms of gender and age distribution, in order to investigate the histology of both their liver tumor and their non-tumor parenchyma.

Some features observed were noteworthy. Over 76% of HCCs were categorized as well to moderately differentiated (G1 and G2), despite the fact that histological assessment was performed on surgical specimens that generally grade higher compared to needle core biopsies^21^. Interestingly, younger HCC patients presented with virtually no fibrolamellar carcinomas, a histotype occurring almost exclusively in non-cirrhotic liver and allegedly earlier than age 40^22–24^. This observation coincides with the results obtained previously in a cohort of 256 Peruvian HCC patients, who were around 40 years old on average and predominantly non-cirrhotic, which yielded only 1.6% of fibrolamellar variant^14^. Furthermore, a significant fraction of the liver tumors scrutinized in the present cohort were steatohepatitic HCC, a relatively rare histological variant that has been associated hitherto with non-alcoholic fatty liver diseases and HCV-related cirrhosis^25,26^.

Data indicate that Peruvian HCC is arising mostly in NTL with low to mild degrees of fibrosis, independently of the tumor histological architecture and grade. Three-quarters of the patients were still in low to moderate Stages 0–2 of hepatic fibrosis, whereas merely one-tenth displayed the severe Stage 3 and eight cases a full-fledged cirrhosis Stage 4 (Table 2). This figure is utterly at odds with the current literature on the topic, as HCC in non-cirrhotic, non-fibrotic livers is generally claimed to represent a small minority of the cases unselected for their NTL features^19,27^.

In order to deepen our understanding of the natural history of the disease, we assessed the levels of fatty liver and steatohepatitis in NTL, as they represent, in some circumstances, a path towards fibrosis and thus enhance the risk of HCC^28–30^. While our results confirmed the low prevalence of severe fibrosis, the fractions of patients with steatosis and steatohepatitis were also relatively low within the cohort, with about a quarter and one-tenth of the patients being affected, respectively (Table 3). These data concur with what was previously described in a large multicenter study pertaining exclusively to HCC occurring in non-cirrhotic livers^31^; whereas our results were obtained within a single-center cohort, unsupervised from a histopathological point of view for the status of the non-tumor liver parenchyma. Above all, multivariate analysis (PCA) did not discriminate NTL features of the Patient Group from those of the Comparative Group, therefore emphasizing the relatively healthy status of the liver in Peruvian HCC patients (Fig. 2a).

It has been reported that parenchymal and parenchymal–mesenchymal-mixed iron overload in NTL is associated with HCC, regardless the cirrhotic status of the liver^32–34^. We thus assessed the level of iron accumulation in NTL from Peruvian HCC patients and compared the results to the data obtained with a comparative group. The very large majority of NTLs were scored with low to moderate grades of iron overload (Grades 0–3+), mostly parenchymal (Table 4). Some degree of iron overload in the liver is relatively common in patients with chronic liver disease, such as alcoholic liver disease and non-alcoholic fatty liver disease^35,36^. Also, iron accumulation has been monitored in the liver of a significant fraction of patients with chronic HCV infection^37^. None of these hepatic affections were observed to be truly prevalent in our cohort of patients; and the mild degree of iron accumulation observed in NTL was not correlated either with fibrosis staging nor fatty liver and steatohepatitis grading of HCC patients. Whether the siderosis monitored in NTL of HCC patients is a confounding factor, a consequence of the liver tumor development, or is related to its etiology remains to be investigated.

When comparing both Patient Group and Comparative Group using discriminant multivariate analysis (OPLS-DA), PAS positive hepatic foci of cellular alteration observed in NTL were strikingly associated with the occurrence of HCC (Figs 2b,c and 3a–e). From a morpho-histological point of view, these hepatic foci are highly reminiscent of liver lesions described hitherto in some animal models that have been exposed to genotoxic chemicals (Fig. 3b–d)^38,39^. This type of liver clear cell foci has also been recently described in human non-cirrhotic liver parenchyma by Ribback and colleagues^40^. However, the authors were not able to assess whether or not, in humans, these lesions were *in fine* precancerous or benign, since they recorded a lower frequency of foci in NTL associated with HCC. Yet, the exact brunt of these hepatic foci of cellular alteration and their alleged role in liver carcinogenesis in animal models has to be elucidated, as well as kinship with what has been observed in human has to be ascertained. Nonetheless, it can be suspected that such foci may have potential to progress to neoplasia, as there is a significant association with HCC as well as some degree of congruence with the co-expression of precancerous marker GS^41^, and thus, that they could be active players in the natural history of the disease in a significant proportion of HCC patients (Fig. 3e).

In order to gain further insights into the burden of these hepatic foci of cellular alteration, we performed a differential gene expression analysis between PAS(+) and PAS(−) NTLs of Peruvian HCC patients. Because hepatic foci represent only a smaller fraction in PAS(+) NTL, there is a strong distortion in disfavor of these foci, lowering the discriminating power of the analysis when directly comparing whole PAS(+) to PAS(−) tissues; so results of such analysis should be viewed cautiously. It is interesting to note, though, some divergent gene expression between NTLs from two sets of Peruvian HCC patients stratified on the presence/absence of these hepatic foci. Indeed, Gene Ontology analysis suggests that the immune system’s activity in PAS(+) NTL is comprehensively modified compared to PAS(−) NTL (Fig. 4a). Concerning olfactory receptors, which are members of a large family of G-protein-coupled receptors also found to be expressed in different non-olfactory tissues, their overexpression has been previously reported to participate in the processes of cancer cell proliferation, metastasis, invasiveness, as well as angiogenesis (Fig. 4b)^42^.

HBV is regarded as a prominent causative agent of HCC, playing an instrumental role in liver carcinogenesis either by oncogene product expression, viral DNA integration into host genes, or through an active viral replication supporting a persistent inflammatory microenvironment that fosters tumor onset^2^. Peruvian HCC develops in an epidemiological context where prevalence of overt chronic HBV infection is intermediate with around 50% of HBsAg carriers and very few HVC-infected cases^6,10,14,43^. This situation implies that a significant fraction of Peruvian patients would have cryptogenic HCC, which is commonly characterized by a well-conserved architecture of the liver parenchyma and absence of a *bona fide* risk factor, such as overt HBV or HCV infections. It has been previously reported that occult HBV infection (OBI), defined by the presence of HBV DNA in blood or liver tissue without detectable levels of HBsAg, can be associated with non-cirrhotic HCC^44^. This is why we undertook a comprehensive molecular detection of HBV in tumor and non-tumor liver tissues. Prevalence of HBV within the cohort was thus initially underestimated as some patients had OBI, disclosing an alternative risk factor for HCC. However, we could not establish a clear-cut correlation between overt and/or occult HBV infection and the occurrence of PAS positive foci of cellular alteration in liver parenchyma, despite the fact that these biomedical features predominate in Peruvian HCC. Given the whole landscape for Peruvian HCC, the potential cooperative role of overt or OBI HBV infection, iron overload, and genotoxic chemicals in liver carcinogenesis etiology has to be further explored, as previously suggested for Chinese HCC cases by Ming and colleagues^45^.

Altogether, our findings uphold the peculiarity of the clinicopathological context of HCC encountered among the patients from South America, especially in Peru^6,13^. It emerges that, in this relatively young patient population, non-cirrhotic, non-fibrolamellar HCC is prevailing. This clinical presentation coincides with the presence of remarkable hepatic foci of cellular alteration in the liver parenchyma that could be the hallmark of liver tissue injury caused by exposure to genotoxic substances^38–40^. In addition, Peruvian HCC displays a unique mutation spectrum for HCC in which short indels are the major class of mutations, what might suggest deficiency in DNA repair mechanisms^10^. It also displays a low rate of alterations in tumor protein p53 (*TP53*) gene and a high prevalence of aberrations affecting genes of the Wnt family [e.g. axin 1 (*AXIN1*) and catenin beta 1 (*CTNNB1*) genes]. The latter are features of a molecular signature reported hitherto for HCC arising in non-cirrhotic livers^46,47^.

Indeed, several studies have already pointed out the occurrence of non-cirrhotic, non-fibrotic HCCs, but not to the extent described in the present study, and with the difference that fibrolamellar carcinomas were herein underrepresented in a relatively young patient population^18,19,22,27,48,49^. According to current views, these non-cirrhotic, non-fibrotic HCCs delineate a distinct nosological entity that displays some noticeable features, all spotted in the clinicopathological context encountered in Peru: (i) a bimodal age distribution with a rather balanced sex-ratio; (ii) an advanced tumor stage at the time of the diagnosis, as it is usually detected due to the onset of cancer-related symptoms with no prodromal period; (iii) relatively moderate distant invasiveness properties; and (iv) a good amenability to liver resection even in cases of major hepatectomy. In addition to this, Trevisani and colleagues specified that the overall and disease-free survivals after resection of these HCCs are equal to those obtained with liver transplantation in cirrhotic patients with early stage tumor, that overall survival depends on HCC burden and is poorly affected by liver function^19^. This information is particularly relevant regarding surgical outcomes in a clinical situation of low- and middle-income countries, where a significant fraction of HCC patients are dismissed from the chain of care due to a poor prognosis empirically determined by canonical staging systems designed elsewhere in an utterly different clinico-epidemiological context^14^.

## Methods

### Ethics approval and consent to participate

Written informed consent was provided by participants for their information and samples to be stored in the INEN Department of Cancer Statistics and Epidemiology (for medical charts) and the INEN Department of Pathology (for tissue specimens) and used for research. When the patient was non-adult, a parent provided the informed consent on his behalf. The present study was carried out in strict accordance with the ethical principles contained in the up-to-date Declaration of Helsinki and was approved by the INEN Human Subjects Committee, protocol numbers #008-2010-CRP-DI/INEN and #113-2014-CIE/INEN.

### Study design and patient selection

The present study was conducted retrospectively within a cohort assembled by analyzing medical charts and surgical specimens of liver resection of 50 patients with HCC (Patient Group) and 75 individuals with primary or secondary, non-HCC liver tumor or benign liver lesions (Comparative Group) who were hospitalized at INEN between October 2012 and August 2017. As a public hospital, INEN accommodates individuals regardless of age, sex, ethnicity, residence, economic status, and health care coverage. Participants were selected on the basis of their pathology report, i.e. whether they had developed intra-hepatic tumors and had undergone surgery. Information on age and gender was checked in order to comply with the clinical epidemiology of HCC patient population in Peru, as previously described^6,10,14–16^.

### Patient management and diagnosis

Patients with malignant liver neoplasms were managed through the INEN Department of Abdominal Surgery. The patients included in the present study were treated by anatomic liver resection, i.e. systematic removal of the tumoral liver segments confined by portal branches to ensure tumor-free margins^14^. After the surgical intervention, pathologists determined primarily tumor size (i.e. longest chord measured), nodule number and presence of macro-vascular invasion, and then assessed the type of cancer cells on hematoxylin–eosin-stained liver sections^50^. In case of HCC, histopathological architecture and grading (G1-G4) were recorded according to the classifications of the World Health Organization and of the American Joint Committee on Cancer, respectively^50,51^. All blood tests were performed at the clinical laboratory of INEN. Tumor marker AFP was monitored for its serum concentration by radioimmunoassay (Roche Diagnostics). Infections with HBV and HCV were monitored in patients’ sera using Elecsys^®^ HBsAg II and Elecsys^®^ anti-HCV II assays (both Roche Diagnostics).

### Histology

Parent NTLs were obtained from tumor-free margins of the resected surgical pieces. Tissues were formalin-fixed, paraffin-embedded (FFPE) in the INEN Department of Pathology according to an established procedure. All tissue staining and immunohistochemistry were performed on the Histo Pathology High Precision (H2P2) platform - certified to ISO 9001 - of the University of Rennes, France. Hepatic steatosis was estimated on hematoxylin–eosin-stained FFPE sections under medium power magnification (x40) and graded (0–3) in accordance with the Non-alcoholic Steatohepatitis Clinical Research Network staging system^52^. Steatohepatitis in NTLs was diagnosed according to the American Association for the Study of Liver Diseases criteria^53^. In addition, possible steatohepatitic HCC variant was assessed using the combination of histopathological features described by Salomao and colleagues^25^. FFPE NTL slices from HCC patients were colored using the Masson’s Trichrome Stain Kit, Artisan™ (Dako), according to the manufacturer’s instructions. Liver fibrosis stage (0–4) was then scored in accordance with the scoring system for fibrosis and cirrhosis described by Scheuer^54^. In parallel, FFPE NTL slices from HCC patients and comparative individuals were stained for the visualization of ferric iron in tissues (Perls’ method) using the Iron Stain Kit, Artisan™ (Dako), according to the manufacturer’s instructions. Histological grade of iron accumulation in NTLs was then scored (0–4+) in accordance with the grading system previously described by LeSage and colleagues^55^. PAS Staining Kit (MilliporeSigma) was used to stain carbohydrates in FFPE NTLs, according to the manufacturer’s instructions (with no diastase treatment). Finally, anti-CD163 (from Abcam), anti-CD34 (from Leica), anti-GS (from BD Biosciences), and anti-smooth muscle actin (from Dako) antibodies were used for immunohistochemistry in FFPE NTLs, conforming to established protocols of the H2P2 platform.

### Transcriptomic analysis

About 50 mg of both HCC and parent NTL matched pair were harvested from the resected surgical pieces, flash-frozen in liquid nitrogen, and stored at - 80 °C (INEN Cancer Research Biobank). Total RNA was later isolated from flash-frozen NTLs using TRI Reagent^®^ (Sigma-Aldrich) and Lysin Matrix D homogenization system (MP Biomedicals), according to manufacturer’s instructions. RNA integrity and quantity were assessed using the RNA 6000 Nano LabChip^®^ Kit (Agilent Technologies) and a 2100 Bioanalyzer System (Agilent Technologies), as well as with the Qubit^®^ RNA Broad-Range Assay Kit (Invitrogen). About 100 ng of total RNA [with RNA integrity number (RIN) >7] were used to evaluate gene expression using GeneChip™ Human Transcriptome Array 2.0 (Applied Biosystems), according to the manufacturer’s instructions. Gene expression analysis was performed using the limma software standard pipeline (R/Bioconductor) at a significance level of 0.05 with no false discovery rate controlling procedure^56,57^. Genes differentially expressed were selected in order to complete a gene enrichment analysis using clusterProfiler software (R/Bioconductor) and the Gene Ontology database (Gene Ontology Consortium) for biological process, with a 0.05 significance level^58^.

### Hepatitis B virus DNA detection

Flash-frozen HCC and NTL samples were pounded under liquid nitrogen and then digested at 37 °C for 8 hours in tissue lysis buffer containing proteinase K and SDS. Total DNA was extracted twice with phenol and once with chloroform, precipitated in ethanol, and resuspended in TE buffer (10 mM Tris; 0.1 mM EDTA; pH 8.0). DNA concentrations were measured using the Qubit^®^ dsDNA Broad-Range Assay Kit (Invitrogen). About 50 ng of total DNA were screened by PCR for presence of HBV DNA, using a nested procedure when appropriate, on at least three different regions of the viral genome, i.e. preS, S, and X/preC. PCRs were carried out with 35 cycles at 95 °C for 1 min, 59 °C for 1 min, and 72 °C for 1–2 min. Subsequently, HBV DNA was quantified by qPCR in 100 ng of total DNA on a CFX96™ Real-Time PCR Detection System (Bio-Rad) using the TaqMan^®^ Pathogen Detection Assay Pa03453406_s1 (Thermo Fisher Scientific). Covalently closed circular DNA (cccDNA) was measured with primers overlapping with the nick of the minus strand, in accordance with Ducroux and colleagues^59^. Amplification specificity for cccDNA was checked by melt-curve analysis. HBV genome equivalent copies were determined on a standard curve generated with known copy numbers of a plasmid containing HBV genome (pFC80). In addition, droplet digital PCRs were performed on a QX100™ Droplet Digital™ PCR System (Bio-Rad) using the TaqMan^®^ Pathogen Detection Assay Pa03453406_s1 and the Human TaqMan^®^ Copy Number Reference Assay (Thermo Fisher Scientific) as a reference. Reaction mixtures consisted in 10 µl of ddPCR™ Supermix (Bio-Rad), 1x primers, and 100 ng of total DNA in a final volume of 20 µl. About 70 µl of Droplet Generation Oil (Bio-Rad) was used to create an emulsion of monodispersed droplets using QX100™ Droplet Generator (Bio-Rad), and emulsified samples were transferred to a 96-well PCR plate. Duplex PCRs were performed on a CM1000 Touch™ Thermal Cycler (Bio-Rad) with the following thermal cycling protocol: denaturation at 95 °C for 10 min, followed by 40 cycles at 94 °C for 30 s with a 2.5 °C/sec ramp rate, 59 °C for 1 min with a 2.5 °C/sec ramp rate, 98 °C for 5 min, and hold at 4 °C. After PCR, 96-well plates were scanned in a QX100 Droplet Reader (Bio-Rad). Data were analyzed using QuantaSoft™ software (Bio-Rad) with autoanalysis settings for duplex experiment.

### Statistics

Medical information was inputted on Microsoft Excel^®^ software version 16.9 (Microsoft Corporation). Statistical tests, i.e. Fisher’s exact test and Student’s *t*-test, were performed two-tailed with a 0.05 significance level using R statistical software version 3.4.3 (R Foundation). One- and two-sided Cochran–Armitage test for trend were performed with Monte Carlo simulation (n = 5,000). For multivariate data analysis, data were imported to SIMCA-P+ multivariate tool version 14.0 (Umetrics) and scaled to unit variance for PCA and OPLS-DA. For OPLS-DA, the model fit was assessed by leave-one-subject-out cross-validation. The validity of the discriminant model was checked using permutation test (Y-scrambling; n = 100).

### Data availability

All data generated or analyzed during this study are included in this published article and its Supplementary Information file, as well as in the NCBI’s Gene Expression Omnibus repository (for microarray data), accessible through GEO series accession number GSE111580 (https://www.ncbi.nlm.nih.gov/geo/query/acc.cgi?acc=GSE111580).

## Electronic supplementary material

Supplementary Information File 1
